# Editorial: Applications of Novel Analytical Methods in Epidemiology

**DOI:** 10.3389/fvets.2018.00243

**Published:** 2018-10-04

**Authors:** Moh A. Alkhamis, Victoria J. Brookes, Kimberly VanderWaal

**Affiliations:** ^1^Department of Epidemiology and Biostatistics, Faculty of Public Health, Health Sciences Center, Kuwait University, Kuwait City, Kuwait; ^2^Department of Veterinary Population Medicine, College of Veterinary Medicine, University of Minnesota, St. Paul, MN, United States; ^3^Sydney School of Veterinary Science, Faculty of Science, University of Sydney, Sydney, NSW, Australia

**Keywords:** spatial epidemiology, social network analysis, risk assessment, disease surveillance, decision making

Over the past decades, the repertoire of quantitative analytical techniques in disciplines such as ecology, decision science and evolutionary biology has grown, in part enabled by the development and increased availability of computational resources. Integration of cutting-edge, quantitative tools into veterinary epidemiology that have been borrowed from such disciplines has offered opportunities to advance the study of disease dynamics in animal populations, to improve and guide decision-making related to disease prevention, control, or eradication. Furthermore, the need to explore new analytical methods for veterinary epidemiology has been driven by the increasing availability and complexity of animal disease data (“big data”). The term “novel” in this research topic indicates methodological approaches that are currently infrequently or previously not used in epidemiology. The objective of this research topic is to contribute to current methods in epidemiology by: (1) presenting and discussing novel analytical tools that help advance our understanding of epidemiology; and (2) demonstrating how inferences emerging from the application of novel analytical tools can be incorporated into decision-making related to animal health.

It is worth noting that traditional analytical methods will continue to be essential tools for guiding and improving disease surveillance because they are computationally less demanding and therefore, more widely accessible. Magro et al. demonstrated the utility of multivariable logistic regression to risk mapping of sinonasal aspergillosis in dogs and in California, whilst Gautam et al. used a Cox proportional hazards model to identify risk factors for culling, sales, and deaths in New Zealand dairy goats.

Identification of suitable environmental and demographic factors for disease outbreaks is an essential component of risk-based surveillance. Escobar et al. offered two unique research articles in which he and his co-authors highlighted the potential of a novel algorithm in ecological niche modeling (ENM) for disease risk mapping of *Toxoplasma, Bartonella*, and *Heterosporis* spp.. They identified important environmental and demographic factors that shaped their predicted spatial distribution, as well as the relative contribution of these factors to this distribution. The two articles set the scene for further development of a powerful analytical approach for predicting disease distribution, thus contributing to the expanding field of spatial epidemiology.

Animal movement between premises has been identified as a critical factor for infectious disease introduction and spread. Valdes-Donoso et al. integrated methods in this research topic by combining data science techniques with social network analysis (SNA) to infer unobserved pig movements between farms. They selected relevant spatial and demographic factors and replicated the structure of a pig movement network from incomplete data by predicting the probability of unobserved movement between sites using supervised machine learning. Their inferences approximated actual unobserved movements between sites; hence this predictive method could guide control strategies between and within geographical regions.

McCabe and Nunn also utilized SNA to offer a unique approach to modeling transmission networks of infectious diseases. They proposed a novel measure for describing a network—“effective network size”—which accounts for the heterogeneity of contacts between the nodes of a given network. They applied this metric to disease outbreak networks simulated from different disease spread models as well as empirical disease networks described in the literature. They found that their metric is highly associated with many traditional network metrics, and hence might provide additional epidemiological insights when using SNA to guide disease intervention.

Assessment of the temporal dynamics of disease outbreaks is another analytical pillar needed to formulate risked-based surveillance systems. Arruda et al. proposed the effective time-dependent reproductive number as a complementary measure of disease spread to use alongside incidence and frequently used spatial analyses such as cluster detection. They demonstrated the utility of computing sequential effective reproductive numbers from case-series data to assess the endemicity of porcine reproductive and respiratory syndrome across regions in the United States. Furthermore, they showed how such a measure could be used to guide and improve intervention strategies to control endemic swine diseases.

Transforming common analytical methods in epidemiology into a Bayesian statistical framework has recently become more common due to the substantial growth in computational resources. Bayesian analytical methods require fewer assumptions about the data than frequentist methods, provide methods to account for uncertainties in data, and can accommodate more complex biological parameters for estimating posterior risk measures of infectious diseases. Gamado et al. developed a toolkit that can be used for risk assessment of epidemic models that are based on discrete-state, continuous-time Markov and semi-Markov processes, using data-augmentation Markov Chain Monte Carlo techniques within a Bayesian framework. They demonstrated how their toolkit could be reliably used to assess risks from potential disease introductions, which subsequently can be used to support and guide prompt disease intervention efforts.

Perception of risk and quantifying preferences among animal producers, health workers, and other stakeholders is another important factor in guiding the implementation of disease intervention efforts. Opinions and perceptions impact policymaking, and thus, advanced analytical methods that can decipher the complexity and diversity of stakeholder preferences are required. Van den Borne et al. borrowed and adapted conjoint analysis (CA) from decision-science. This method has had limited use in veterinary science, with applications previously focused on disease prioritization. Van den Borne et al. used a computer-based adaptive choice-based conjoint analysis to elicit respondents' preferences for design characteristics of a new udder health and antimicrobial usage improvement program in Switzerland. They demonstrated the novelty and advantages of their approach in guiding decision-makers to both administer current animal health programs and develop new programs based on the independent opinions of veterinarians and animal producers.

In this research topic, a selected array of novel analytical methods from a variety of scientific disciplines was demonstrated, with the goal of complementing and advancing the field of epidemiology. Our intention is that this research topic further motivates epidemiologists to seek and develop analytical methods to overcome current analytical limitations; application of novel and interdisciplinary methods new to the field of veterinary epidemiology has the potential to expand the horizons of veterinary epidemiological research.

## Author contributions

All authors listed have made a substantial, direct and intellectual contribution to the work, and approved it for publication.

### Conflict of interest statement

The authors declare that the research was conducted in the absence of any commercial or financial relationships that could be construed as a potential conflict of interest.

